# Limitations of the Use of Pressure Waves to Verify Correct Epidural Needle Position in Dogs

**DOI:** 10.1155/2013/159489

**Published:** 2013-06-18

**Authors:** Chiara Adami, Alessandra Bergadano, Claudia Spadavecchia

**Affiliations:** ^1^Department of Veterinary Clinical Science, Anesthesiology and Pain Therapy Division, Vetsuisse Faculty, University of Berne, Länggasstraße 124, 3012 Berne, Switzerland; ^2^Comparative Medicine Division, F. Hoffmann-La Roche AG, Grenzacherstraße 124, 4070 Basel, Switzerland

## Abstract

The use of pressure waves to confirm the correct position of the epidural needle has been described in several domestic species and proposed as a valid alternative to standard methods, namely, control radiographic exam and fluoroscopy. The object of this retrospective clinical study was to evaluate the sensitivity of the epidural pressure waves as a test to verify the correct needle placement in the epidural space in dogs, in order to determine whether this technique could be useful not only in the clinical setting but also when certain knowledge of needle's tip position is required, for instance when performing clinical research focusing on epidural anaesthesia. Of the 54 client-owned dogs undergoing elective surgeries and enrolled in this retrospective study, only 45% showed epidural pressure waves before and after epidural injection. Twenty-six percent of the animals showed epidural pressure waves only after the injection, whereas 29% of the dogs showed epidural pressure waves neither before nor after injection and were defined as false negatives. Our results show that the epidural pressure wave technique to verify epidural needle position lacks sensitivity, resulting in many false negatives. As a consequence, the applicability of this technique is limited to situations in which precise, exact knowledge of the needle's tip position is not mandatory.

## 1. Introduction

Locoregional anaesthesia is becoming increasingly popular in veterinary medicine. Among the different techniques, epidural administration of local anaesthetics and analgesics is nowadays widely employed in canine clinical patients, especially when performing orthopedic procedures involving the hind limbs [1, 2]. Beside its clinical application, within the last decade, epidural anaesthesia has been the focus of a large number of experimental investigations [3–5]. 

A proper needle placement into the epidural space is essential to correctly perform the technique and also to forecast the likelihood for the epidural injection to result in successful analgesia. Confirmation of the proper needle positioning is useful in the clinical setting and mandatory when performing clinical studies in which the epidural injection plays a central role. 

Several techniques have been described to verify the correct needle placement into the epidural space. Traditionally, the “pop sensation,” the “hanging drop” (HD), and the “loss of resistance” techniques have been used in the clinical setting [6–8]. These methods have the advantage of being inexpensive; however, they are based on subjective perceptions, which can make them unsuitable for being used for experimental or clinical research. More objective techniques have been developed in the last decade. Contrast radiography, fluoroscopy, electrical stimulation of the spinal cord through a nerve stimulator, and ultrasound-guided spinal needle placement have been described in humans and dogs [9–14]; however, some of these techniques are often poorly applicable due to the need of specialized equipment, and all of them present several limitations. Contrast radiography and fluoroscopy are considered gold standard methods for verifying needle position; however, they are expensive and time consuming. Furthermore, the administration of the contrast media might result in allergic reactions [15]. Ultrasound-guided epidural needle and catheter placement is commonly performed in children [9, 10] but is technically difficult and requires a certain degree of expertise in ultrasonography. The specificity and sensitivity of the epidural electrical stimulation method have been investigated in dogs [11, 12] however, this technique elicited false-positive motor responses in pigs, when the needle was not yet in the epidural space and resistance to injection was detected [13].

The use of pressure waves to confirm the correct position of the spinal needle has been described in humans, dogs, horses, goats, and cattle [13, 15–21]. Potential advantages offered by this promising technique are objective and have easily interpretable outcomes and considering that nowadays many hospitals have at their disposal multiparametric monitors capable of measuring pressures via a pressure transducer, the fact that sophisticated additional equipment is not needed. 

The aim of this retrospective study was to evaluate the sensitivity of the pressure waves as a method to objectively verify the position of the needle in the epidural space of dogs, in order to assess the usefulness and the applicability of this technique in those situations, such as clinical and experimental research focusing on epidural anaesthesia, in which reliable confirmation of the needle position is essential. 

Our hypothesis was that this method, used as a test for correct needle placement, may result in the observation of many false negatives, thus offering low sensitivity.

## 2. Methods 

Fifty-four client-owned dogs scheduled for stifle surgeries and included in clinical trials other than this for which ethical permission of the local authority (license number 41/10) and signed informed owner consent were obtained were enrolled in this retrospective study. For these patients a single lumbosacral epidural injection was selected to provide intraoperative analgesia, and pressure wave recording was used to verify the correct epidural needle position. Only records of animals classified as American Society of Anaesthesiologists (ASA) I or II and which fulfilled the criteria for correct needle positioning (observance of positive response to the HD test prior to epidural injection, loss of anal responsiveness to suture, decrease in mean arterial pressure or heart rate after injection, and presence of residual motor block during recovery phase) were included in this study. During preanaesthetic examination, body weight (kg) was measured and a BCS [21] assigned to each dog and recorded. Dogs were premedicated intramuscularly (i.m.) with either a combination of acepromazine (0.01 mg/kg; Prequillan, Fatro, Italy) and methadone (0.2 mg/kg; Methadone, Streuli AG, Switzerland), or acepromazine only (0.03 mg/kg). The skin was aseptically prepared and an intravenous (i.v.) catheter was placed percutaneously in the cephalic vein. Twenty minutes after premedication, general anaesthesia was induced with i.v. propofol (Propofol, Fresenius, Switzerland), titrated to effect. The trachea was intubated with an appropriate size endotracheal tube, and then isoflurane (Isoflurane, Abbott, USA) in air/oxygen (1 : 1) was delivered via a circle breathing system. The dogs were fully monitored and physiologic variables manually recorded at intervals of 5 minutes. A constant end-tidal isoflurane concentration of 1.3%, equivalent to the minimum alveolar concentration [22], was targeted during anaesthesia. A balanced crystalloid solution was administered i.v. at the rate of infusion of 10 mL/kg/h. Dogs were allowed to breath spontaneously unless the end tidal carbon dioxide reached more than 45 mmHg; if that occurred, pressure-supported ventilation with a peak inspiratory pressure of 10 cmH_2_O was performed to maintain the end-tidal carbon dioxide lower than 50 mmHg. The dorsal metatarsal artery was catheterized with a 20 G cannula to allow the continuous measurement of the systolic, mean, and diastolic blood pressure.

After a stable anaesthesia level was achieved, the dogs were positioned in sternal recumbency with the hind limbs pulled forwards symmetrically to maximise the dorsal lumbosacral space. Wings of the ilium, the dorsal spinosus processes of L6, L7, and the sacrum were used as anatomical landmarks. After surgical preparation of the area, a 75 mm 19-gauge spinal needle was placed percutaneously through the intervertebral ligament between L7 and S1 into the epidural space, with the bevel facing cranially. The epidural puncture was always performed by an experienced anaesthetist. The needle was slowly advanced and, when the typical “pop” sensation was perceived, this was considered indicative of piercing the *Ligamentum flavum*; at this point, the HD technique was used to confirm the correct needle position. The pressure measuring system was set up as previously described by Iff and colleagues [15] as follows: the spinal needle, once inserted in the epidural space, was connected to a sterile, fluid-filled, nondistensible pressure line; the latter was connected to a pressure transducer (Angiokard; Medizintechnik, GmbH & Co.), in continuity to both a continuous flush device and to the multiparametric monitor (Kion SC7000, Siemens, Germany), and placed at the level of the transverse process of the last lumbar vertebra for zeroing. The continuous flushing device consisted of a 250 mL NaCl 0.9% in a pressurized bag (33 kPa), set to deliver 2.5 mL/hour. The presence or absence of epidural pressure waves (EPW) was noticed, and the mean baseline pressure values were measured and recorded after an equilibration period of three minutes. When the recorded pressure values were below 0.7 kPa, identification of the pressure waves was facilitated by setting the lowest pressure scale (1.3 kPa) on the monitor. The fluid-filled nondistensible line was shortly disconnected to allow the connection of the syringe to the spinal needle for the epidural injection. The injected volume was 0.2 mL/kg for each animal, given manually as a single bolus delivered over 2 minutes. Twenty-one dogs received 0.5% ropivacaine, whereas 19 dogs received a combination of 0.5% ropivacaine, 1 *μ*g/kg sufentanil, and 0.9% NaCl (to dilute the ropivacaine to a concentration of 0.25%), and 14 dogs received a mixture of 0.5% ropivacaine, 1 *μ*g/kg sufentanil, 6 *μ*g/kg preservative-free epinephrine, and 0.9% NaCl. Immediately after the epidural injection, the fluid-filled line was reconnected to the spinal needle. The presence or absence of postinjection EPW was noticed, and the postinjection pressure values were measured and recorded. The increase in epidural pressure (Δ*P*) was calculated as the difference between the baseline pressure and the pressure recorded immediately after the injection. Five minutes after the end of the epidural injection, the following categorical variables were recorded: the loss of anal responsiveness to suture (yes or no) and decreases in mean arterial pressure or heart rate more than 30% of the values recorded prior to injection within 20 minutes from the end of epidural injection (yes or no). 

At recovery, as soon as the animals were able to attempt standing, they were evaluated for the presence of residual motor block and hind limb weakness. 

The observance of a loss of anal responsiveness to suture and of a decrease in mean arterial pressure or heart rate below the defined values after injection, together with the observance of positive response to the HD test prior to injection and with the presence of residual motor block during recovery phase, was considered indicative of successful epidural injection and correct needle position; therefore, dogs which fulfilled all these conditions but showed EPW neither prior to nor after epidural injection were considered false negatives. Dogs that did not fulfill these requirements were excluded from the study. 

Statistical analysis was performed using commercially available software (NCSS Statistical Software 2007, UT, USA; and SigmaStat 2011, Systat Software Inc., CA, USA); *P* values < 0.05 were considered statistically significant. 

To assess whether data were normally distributed, Shapiro-Wilk test was used. Spearman correlation coefficient was used to determine the association between BCS and Δ*P*, body weight and Δ*P*, and age and Δ*P*. 

Correlation between the presence of pressure waves (before and after epidural injection) and decrease in mean arterial pressure below the cut-off value was determined with Fisher's exact test. 

Correlation between the other variables (presence of EPW prior to injection versus decrease in mean arterial pressure, response to HD, presence of residual motor block at awakening, and loss of anal responsiveness to suture; and presence of EPW after injection versus all the above-listed variables) was determined by using *χ* ^2^ test. 

Kruskal Wallis one-way analysis of variance was used to evaluate the differences in Δ*P* and in the pre- and postinjection pressure values, between the dogs receiving a combination of methadone and acepromazine in premedication (group AM) and those receiving acepromazine only (group A). 

A 2 × 1 table (Table 1) was used to calculate the sensitivity of the EPW test for verifying the correct epidural needle position in comparison with the reference technique; the latter was based on the fulfillment of all the clinical conditions, indicative of correct needle placement, previously described. 

## 3. Results

The results are reported as mean (sd) values for the normally distributed data and as the median and the range (min-max) for data which were not normally distributed. 

The dogs enrolled in this study had a mean body weight of 35.5 (±16.4) kg and a mean age of 5.5 (±2.7) years. Median body condition score (BCS) was 3 (range 2.5–4.5). Twenty-seven dogs were females. Surgery lasted 120 minutes (80–180). Twenty-one animals received a combination of acepromazine and methadone (group AM) in premedication, whereas the other 33 received acepromazine only (group A). 

Mean preinjection epidural pressure was −0.3 (±0.9) kPa, whereas mean pressure value recorded after epidural injection was 3.4 (±2.8) kPa. The mean difference between pre- and postinjection pressure values (Δ*P*) was 3.5 (±2.3) kPa. Prior to injection, subatmospheric epidural pressure values were recorded in 43 dogs only. 

**Figure 1:**
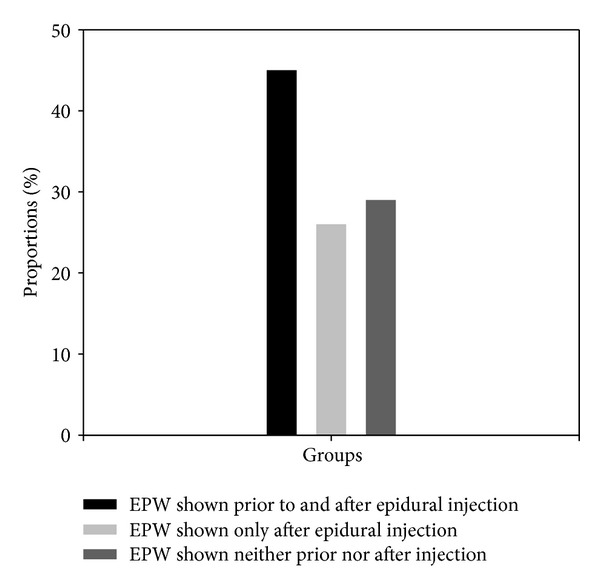
Proportions of dogs showing epidural pressure waves prior to and after the epidural injection (45%), only after the epidural injection (26%), and neither prior to nor after injection (29%).

**Figure 2:**
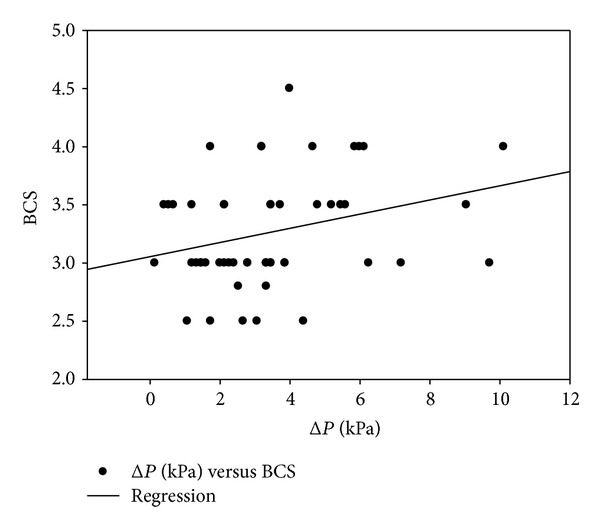
Correlation between body condition score (BCS) and the difference between baseline and postinjection epidural pressures (Δ*P*); Spearman correlation coefficient = 0.3; *P* = 0.038.

**Figure 3:**
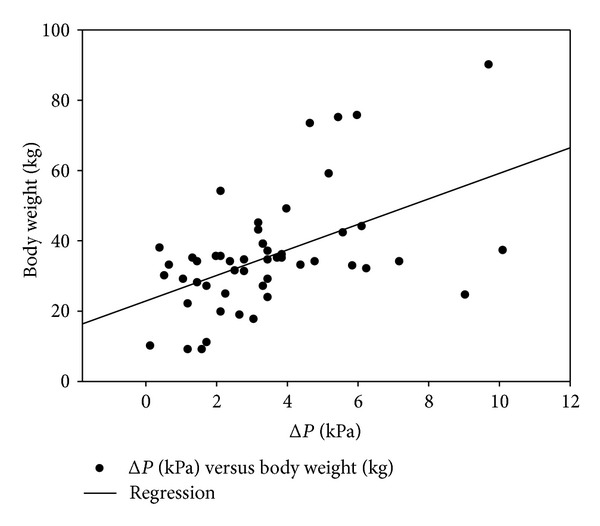
Correlation between body weight in kg and the difference between baseline and postinjection epidural pressures (Δ*P*); Spearman correlation coefficient = 0.48; *P* = 0.0009.

Only 45% of dogs (*n* = 24) showed epidural pressure waves (EPW) before and after epidural injection. Twenty-six percent of the animals (*n* = 14) showed EPW only after the epidural injection, whereas 29% of dogs (*n* = 16) showed EPW neither before nor after injection and were defined as false negatives (Figure 1). The sensitivity of the EPW technique in comparison to the reference technique was 70% (Table 1). Statistically significant correlations were found between BCS and Δ*P* (Spearman coefficient: 0.3;  : 0.038; Figure 2) and between body weight and Δ*P* (Spearman coefficient: 0.48; *P* = 0.0009; Figure 3). 

No significant correlations were found between the other categorical variables. 

**Figure 4:**
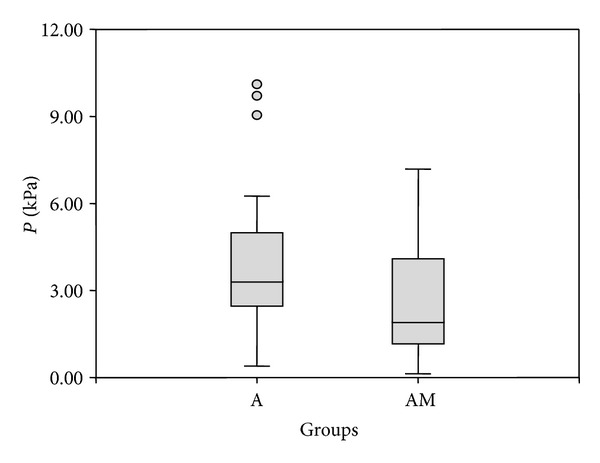
Difference between pre- and postinjection epidural pressure values (Δ*P*) in dogs receiving only acepromazine in premedication (group A) and dogs receiving a combination of acepromazine and methadone (group AM). The box and the line represent the interquartile range and the median, respectively; the whiskers indicate minimum and maximum. The difference in Δ*P* between the two groups is statistically significant (*P* = 0.03).

No statistically significant differences in pre- and post- injection pressure values were found between group A and group AM; however, in group AM Δ*P* was significantly lower than in group A (*P* = 0.03; Figure 4). 

Breed distribution in the canine population enrolled in the study is summarized in Table 2.

## 4. Discussion 

Our results show that the detection of EPW poorly correlates with the more traditionally employed hanging drop (HD) technique, as well as with the observance of the classical effects of the epidural administration of local anaesthetics, such as decrease in arterial blood pressure and heart rate, loss of anal responsiveness to suture, and residual motor block after awakening. This is in contrast with the findings of a recent study [23] in which the investigators identified the EPW in 89% of dogs with successful epidural puncture, although in 35% of these dogs, the EPW occurred following extradural injection but not before. 

Considering that only dogs with positive HD test were included in this study, we expected the epidural pressures recorded prior to injection to be subatmospheric in all subjects. However, this was not the case. The occurrence of positive pressures recorded in some dogs prior to injection may be due to a caveat of the measurement technique: because the needle was connected to the pressure transducer only after performing the HD test, during this time the epidural space was exposed to the atmosphere and it is possible that the extradural and the atmospheric pressures equilibrated, contributing to render the first one less negative. Furthermore, the HD itself, together with the small amount of fluids delivered by the continuous flushing device, could have further increased the extradural pressure before the values could be obtained and recorded. 

As an alternative explanation, the positive pressure values recorded in some dogs might have been the result of an extension of the spinal cord caudally to the lumbosacral junction in these subjects, leading to penetration of the needle into the intrathecal space or even into the spinal cord. However, considering that such a caudal extension of the spinal cord is more likely to occur in young or small-sized dogs and that the animals in which positive pressure values were recorded were all adult and had a body weight ranging from 20 to 29 kg (minimum and maximum, resp.), this explanation seems to be unlikely.

Although this is in contrast with previous findings [15], in the present study, a correlation was found between body weight and Δ*P* and between BCS and Δ*P*. A reasonable explanation for this observation could be the presence of a considerable amount of epidural fat in obese dogs which, by reducing the epidural space, may facilitate a rapid rise in pressure after injection. 

However, it should be noticed that, as dogs of different sizes were enrolled in the study, the clinical significance of the correlation between body weight and Δ*P* may be debatable. 

The abundance of epidural fat in obese patients might also compromise the observance of EPW, as the adipose tissue could act as a buffer and blunt the pressure oscillations within the epidural space; nevertheless, no correlation between occurrence of EPW (pre- and postinjection) and BCS was found. 

Different anaesthetic protocols could affect the pre- and postinjection epidural pressures by inducing cardiovascular changes directly or indirectly influencing the dynamic of the cerebrospinal fluid. Acepromazine, which was used at two different doses to premedicate the dogs included in this study, can cause a clinically relevant decrease in arterial blood pressure resulting from blockade of alpha 1 receptors adrenergic in the peripheral vasculature. However, in this study, higher doses of acepromazine did not result in lower epidural pressure values; on the contrary, group AM had lower Δ*P* values than group A, which received the greatest dose of acepromazine. 

The different epidural drug combinations that were used in this study might have influenced our results: compared to dogs in which ropivacaine alone was injected, in dogs receiving epidural drug mixtures, the dilution of the local anaesthetic could have led to attenuation of some of the clinical indicators of correct needle placement, such as motor block and loss of anal response. However, the exclusion from the study of the animals that did not fulfill the previously listed requirements should have contributed to overcoming this inconvenience. 

In many dogs, EPW could be detected only after epidural injection. It is hypothesized that the pressure oscillations within the epidural space become more evident if the epidural pressure increases, which occurs after volume injection. The observance of postinjection EPW in dogs not showing them prior to injection could still be interpreted as a confirmation of successful needle placement; however, deciding to perform the epidural injection despite the absence of objective evidence of correct needle position carries the risk of failure in performing the technique appropriately. 

The exclusion from the study of the subjects in which the accuracy of the epidural needle placement could not be confirmed implies a lack of true negatives. In order to include the true negatives in the study, beside the EPW recording, we should have performed a test capable of reliably detecting the incorrect epidural needle positioning, namely, radiographic exam or fluoroscopy; however, because this was not designed as a prospective investigation, these tests were not performed. Another reason not to perform additional techniques beside pressure waves was the need for proposing a protocol applicable to client-owned dogs in terms of ethical requirements: radiographic exam and fluoroscopy are time consuming and expensive and would have considerably prolonged the duration of anaesthesia as well as the costs for the owner. 

The lack of a radiographic confirmation of the needle position in the epidural space is one limitation of this retrospective analysis and implies that the specificity of the EPW test cannot be evaluated in this study. In order to overcome this bias, we included in the study only the dogs in which all clinical signs that could be interpreted as indicators of properly performed epidural injection, such as loss of anal responsiveness to suture, decrease in mean arterial blood pressure or heart rate, and residual motor block at recovery, were observed. The occurrence of all these markers in each animal enrolled in the study was considered indicative of correct needle position and used as positive control. 

## 5. Conclusion

The high proportion of false negatives detected in this study bears out our hypothesis and indicates that the EPW technique for verifying the correct needle position in the epidural space has low sensitivity. This drawback discourages its use in those situations in which the confirmation of the epidural needle position plays a central role, as it is the case for clinical and experimental research. 

Nevertheless, it should be mentioned that the EPW technique is inexpensive, easy to perform, and not time consuming; these advantages could make it suitable for a purely clinical use and in general when certain knowledge of the exact epidural needle position within the epidural space is not essential. 

## Figures and Tables

**Table 1:** 2 × 1 table used to calculate the sensitivity of the epidural pressure waves recording in comparison with the standard technique. The epidural pressure waves' test sensitivity was determined by the number of positive subjects divided by the total number of subjects in which, according to the standard technique, the epidural needle was successfully placed.

	Correct needle position (number of subjects)
Positive	38
Negative	16

Total	54

**Table 2:** Breeds distribution in the canine population object of the study.

Breed	Number of dogs
Mixed breed	12
Labrador retriever	5
Golden retriever	4
German shepherd	4
Bernese mountain dog	3
Doberman	3
Vizsla	1
Boxer	3
Newfoundland	2
Saint Bernard	2
Tervueren	1
English bulldog	1
Siberian husky	1
Great Swiss mountain dog	4
Swiss mountain dog	4
German shorthaired pointer	4
